# A Pilot Study Examining the Association of Parental Stress and Household Food Insecurity with Dietary Quality in Pre-School-Aged Children

**DOI:** 10.3390/nu15143154

**Published:** 2023-07-14

**Authors:** Madison McCarthy, Mara Z. Vitolins, Joseph A. Skelton, Edward H. Ip, Callie L. Brown

**Affiliations:** 1 Wake Forest University School of Medicine, Winston-Salem, NC 27157, USA; 2 Department of Epidemiology and Prevention, Wake Forest University School of Medicine, Winston-Salem, NC 27157, USA; mvitolin@wakehealth.edu; 3 Department of Pediatrics, Wake Forest University School of Medicine, Medical Center Boulevard, Winston-Salem, NC 27157, USA; jskelton@wakehealth.edu; 4 Department of Biostatistics, Wake Forest University School of Medicine, Winston-Salem, NC 27157, USA; eip@wakehealth.edu

**Keywords:** children, parents, stress, household food insecurity, dietary quality, diet

## Abstract

Adequate dietary quality is necessary for children’s appropriate development and may be influenced by family factors. This study with 24 healthy 3–5-year-old children assessed the associations of parental stress and household food insecurity (HFI) with a child’s dietary quality. Parents completed three 24 h dietary recalls, and the Healthy Eating Index was calculated to assess dietary quality. Parents also completed a questionnaire, including The Perceived Stress Scale (assessing overall parental stress) and the Hunger Vital Sign screen (assessing HFI). Children’s height/weight were measured, and BMIz was calculated. Separate multivariable linear regression models assessed the association of dietary quality components with HFI and parental stress, adjusting for household income, child sex, and child BMI z-score. In bivariate analyses, children with HFI consumed more added sugars, and parental stress was associated with the child’s greens/beans intake. In multivariable analysis, HFI was associated with lower total protein scores and higher added sugar intake, while parental stress was associated with lower greens/beans intake. Higher household income was associated with higher total vegetable and sodium intake, and children with a higher BMIz had a lower total protein intake. Parental stress and HFI can impact a child’s dietary quality; providers should counsel families on strategies to improve diet quality.

## 1. Introduction

A well-rounded, healthy diet is needed for the proper development of a child. Dietary quality can be measured by determining the variety and adequacy of food groups. Previous research has evaluated the dietary quality of children ages 2–19 who live in the United States and found that only 1% of these children met the national recommendations for dietary variety [1]. Specifically, most children will consume more than the recommended fats and sugars while not eating enough fruits, grains, or dairy [1,2,3]. The impact of dietary variety on a child’s physical health has subsequently been evaluated. Through extensive systemic review, it has been noted that decreased dietary variety in young children aged <5 has longitudinal effects, with the strongest association between low dietary diversity and growth stunting seen later in development and less of an established association with low dietary diversity and wasting and being underweight [4]. Researchers have identified several influences on a child’s diet, including but not limited to the quality of the mother’s diet, the availability of certain foods in the home, household income, food insecurity, and familial stress [5,6,7,8,9,10]. Household food insecurity (HFI) is defined as inadequate access to necessary food to feed the members of a household because of a lack of resources [11]. In 2020, it was noted that 10.5% of US households were food insecure and 14.8% of households with children were food insecure, which was an increase from the year prior [11]. It has previously been reported that children of all ages who experience HFI are at a higher risk of developing chronic illnesses and have poorer health outcomes [12,13,14]. Importantly, children with HFI are at a higher risk of developing disordered eating, asthma, depressive symptoms, and an overall poorer health status [12,13,14]. Furthermore, children with HFI are found to have more frequent headaches, stomachaches, and upper respiratory illnesses, along with higher rates of emergency department visits [13,14]. Moreover, previous research regarding food insecurity and dietary quality has provided varying results depending on whether the diet of the child or adult was examined, with one study noting an adverse relationship between food insecurity and dietary quality in adults but a less consistent relationship seen in children with food insecurity [15]. In this study, it was determined that, compared to adults with food security, food-insecure adults consumed less vegetables, fruits, and dairy, along with a decrease in the intake of certain vitamins and minerals. For children with food insecurity evaluated in this study, it was reported they only had lower fruit consumption compared to children with food security [15]. This difference between adults and children warrants further evaluation.

A recent review confirmed that most studies associate food insecurity with the worsening mental health of parents, such as parent depression, anxiety, and stress [16]. Parental stress, which has been documented as prevalent in up to 29% of households, can also impact a child’s dietary variety [17]. One study noted that children in low-income households with documented parent-perceived stress had increased rates of obesity and increased fast food consumption [18]. Further, it has been noted that family stress can ultimately result in an overall negative influence on a child’s diet [19]. With the increased intake of fast foods, these children will consume additional saturated fats and salt, which puts them at a higher risk for developing cardiovascular disease later in life [19]. While the relationships between food insecurity and parental stress have been evaluated in separate cohorts, it is unclear how these factors affect a child’s dietary quality. Therefore, this study will evaluate the effects of parental stress and household food insecurity together on a child’s dietary quality.

## 2. Methods

### 2.1. Study Overview

Twenty-four parent–child dyads were recruited via the phone from a larger study where three hundred and thirty-five participants were recruited from one of four pediatric primary care clinics. Parents of the 3–5-year-old children completed three 24 h dietary recalls by phone during two weekdays and one weekend day; the nutrient composition of these was averaged. The Healthy Eating Index (HEI) was used to assess the overall dietary quality, which considers 10 components: grains, vegetables, fruits, milk, meat, saturated fat, total fat, cholesterol, sodium, and added sugars [20]. The Perceived Stress Scale (PSS) assessed parental stress, and the 2-item Hunger Vital Sign screen (HFI) assessed household food insecurity. Children’s height and weight were measured, and their BMIz score was calculated. This study was approved by the Wake Forest University School of Medicine Institutional Review Board. 

### 2.2. Participants

This study assessing the dietary quality of 24 pre-school-aged children was part of a larger study that examined the feeding practices of healthy 3–5-year-old children [21]. Parents and their children were enrolled in the larger study when they were visiting one of four general pediatric clinics associated with Atrium Health Wake Forest Baptist for a routine healthcare appointment. The clinics provide care for diverse patients and include an urban clinic, an urban pediatric residency continuity clinic, a suburban clinic, and a rural clinic. Inclusion criteria for the parents included being ≥18 years old and able to read and write in English. Children were excluded if they were born prematurely (<34 weeks gestation), had a birth weight <2500 g, had a developmental or intellectual disability, or had a medical condition that impacted their weight or diet. Children with documented allergies to food or medications were also not eligible for the larger study. Families were recruited sequentially, and only one child per family was included in the study. If more than one eligible sibling was in the clinic together, the parent was asked about the participation of the younger sibling. After parents provided informed, written consent, parents completed a survey in the examination room, either on paper or using a tablet with REDCap. Parents were given the option to have the survey read to them to address low literacy; however, no parents requested this option. The survey included the Child Feeding Questionnaire’s (CFQ) Pressure to Eat subscale [22,23]. The responses, which ranged from 1–5, were averaged, with higher scores indicating a higher pressure to eat. Respondents were then classified as high pressure (mean subscale score >3; n = 106) or low pressure (mean subscale score <2; n = 68); some participants scored in the mid-range (2–3) and therefore were not classified as either high or low pressure. 

Parent–child dyads who completed baseline measures at recruitment and who were classified as either high or low pressure based on their CFQ subscale score were invited to participate in this part of the study, which consisted of additionally completing dietary recalls (used in this analysis) and participating in a standardized feeding assessment. All eligible parents (n = 174) were contacted via phone sequentially: 78 participants were not able to be contacted, 45 declined participation due to work/school schedules, 26 canceled their involvement, and 1 consented but did not complete the dietary recalls; thus, 24 dyads were successfully recruited, with 12 parents self-reporting high pressure to eat and 12 parents self-reporting low pressure to eat. To be able to detect differences in stress during mealtimes by pressure-to-eat status, we selectively recruited participants to have both high-pressure-to-eat behaviors and low-pressure-to-eat behaviors represented in our small pilot sample. 

### 2.3. Measures

The Perceived Stress Scale (PSS) was used to evaluate parental stress. This 10-item scale assesses how uncontrollable, unpredictable, and overloaded participants find their lives. Responses are summed, and a higher score indicates a higher level of stress [24]. The PSS has high internal consistency (α = 0.89), and test–retest reliability scores range from 0.55 to 0.85.

To assess HFI, the 2-item screen by Hager et al. was used [25]. A positive screen is noted as an affirmative response to either of the two questions. This screening measure has high sensitivity (97%) and specificity (83%) when compared to the gold-standard 18-item USDA Household Food Security Survey [26].

Parents completed three 24 h dietary recalls over the phone on two weekdays and one weekend day and the results were used to assess the child’s dietary intake. During these calls, which were administered by the Bionutrition team from the Clinical Research Unit, the parent was asked to recall all of the foods and beverages consumed by the child during the 24 h prior to the interview. To ensure complete dietary recall, parents were instructed to ask the appropriate caregiver of the child if the child was with a babysitter, in school, or pre-school at any time. The Nutrition Data System for Research (NDSR) software version 2020, developed by the Nutrition Coordinating Center, University of Minnesota, Minneapolis, MN, was used to collect and analyze the dietary intake data. Using NDSR, the nutrition composition was calculated using the mean of the three recalls at each time point. To determine the frequency and amount of consumption, foods and drinks consumed were tabulated. The Healthy Eating Index (HEI-2015) was then calculated to assess overall dietary quality, which considers 10 components (grains, vegetables, fruits, milk, meat, total fat, saturated fat, cholesterol, sodium, and added sugars) [20].

Parents also self-reported demographic information such as parents’ income (<USD 20,000, USD 20–39,999, USD 40–59,999, USD 60,000 or more) and education level (less than high school, high school graduate, some college, associates/bachelors/masters/or higher). The child’s biological sex, race, and ethnicity (categorized in this study as Hispanic, non-Hispanic black, non-Hispanic white, or other for this study) and the number of members in the household were also recorded. During the patient’s clinic visit, the child’s height and weight were measured by nursing staff using a wall-mounted stadiometer and mechanical beam scale and recorded in the clinical record. Clinical measurements have been shown to have good accuracy when compared to height and weight measurements obtained for research purposes [27]. Body mass index z-scores (BMIz) were calculated, and BMI percentiles were determined using the Centers for Disease Control and Prevention reference growth charts for age and sex [28]. Weight status was classified as underweight (BMI < 5th percentile for age and sex), healthy weight (5th to <85th percentile), overweight (85th to <95th percentile), and obesity (≥95th percentile). 

### 2.4. Statistical Analyses

Two sample T-tests were used to assess differences in dietary quality components by HFI status. Unadjusted linear regression assessed differences in dietary quality components based on parental stress. Separate multivariable linear regression models assessed the association of dietary quality components, including the overall HEI score and the moderacy and adequacy subscales and their components, with HFI and parental stress. Analyses were adjusted for household income, child sex, and child BMI z-score, which were chosen a priori based on the literature. All analyses were performed using Stata/SE v. 14.2 (College Station, TX, USA: StataCorp LP).

## 3. Results

The 24 parent–child dyads were racially and ethnically diverse, with 42% Black, 42% White, and 17% Hispanic, and 53% of the children were male. Socioeconomic diversity was represented, with participants having a wide range of incomes, including 33% making less than USD 20,000, 29% USD 20,000–USD 60,000, and 38% with USD 60,000 or greater. Parents were 96% mothers, and 66% had obesity. Household food insecurity was recorded in 12.5% of families. Children had an overall low dietary quality, with a mean HEI score of 55.2 (SD 10.9) (Table 1). At least two dietary recalls were completed for the 24 children included in the analysis; 20 completed all 3 recalls.

**Figure 1:**
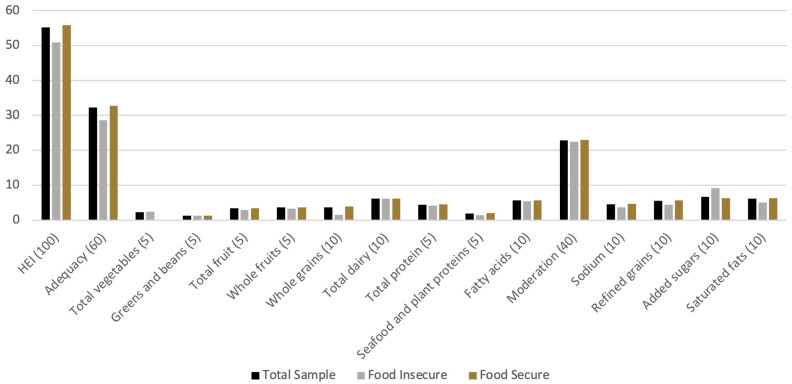
Dietary Quality Component (with Maximum Possible Points) by Food Insecurity Status. I uploaded a new version of the figure without this title.

In bivariate analysis, children with HFI had an overall HFI score that was 4.8 points lower (poorer quality) than children with food security (Figure 1), although this difference was not statistically significant (Table 1). Children with food insecurity consumed more added sugars (9-point unadjusted difference, *p* = 0.04, effect size 1.26) and fewer whole grains (2.4-point unadjusted difference, *p* = 0.3, effect size 1.04). Parental stress was also not significantly associated with overall dietary quality; however, it was associated with vegetable intake, such that for each 1-point increase in the PSS, the child’s total vegetable score was 1.6 points lower (*p* = 0.01).

In multivariable analysis, children with HFI had a 6.7-point lower overall dietary quality score (Table 2). HFI was associated with a lower total protein intake and a higher added sugar intake for children. For each 1-point increase in the parental stress score, the child’s overall dietary quality was 3.9 points lower, although this difference was not statistically significant. Parental stress was significantly associated with the child’s overall lower greens and beans intake (Table 2). Additionally, higher household income was associated with higher total vegetable and sodium intake, and children with a higher BMIz had a lower total protein intake (Table 2).

## 4. Discussion

In this sample of 24 racially and socioeconomically diverse parent–child dyads, it was found that children in households with food insecurity consumed significantly less protein and more added sugar. Lower consumption of greens and beans was significantly associated with increasing parental stress. While children with HFI had a 6.7-point lower overall dietary quality score and PSS was associated with a 3.9-point lower overall dietary score, these differences were not statistically significant. Higher household income was associated with higher total vegetable and sodium intake, and a higher BMIz was associated with lower protein intake.

Our findings regarding the impacts of parental stress and HFI on a child’s dietary quality contribute to the growing body of literature on the social factors that influence a child’s health. Previous studies in children have shown an overall HEI SD of 10–11, similar to the SDs found in our study [29]. Applying an effect size of 0.5 (consistent with a moderate effect), a difference of 5–6 points in the overall HEI score is typically considered a meaningful difference in overall dietary quality. While not statistically significant, the differences in overall dietary quality caused by food insecurity and parental stress in our study have effect sizes that are potentially clinically meaningful and should be further investigated in future studies with larger sample sizes.

Prior research has demonstrated that parental stress may adversely impact a child’s dietary variety, and this decrease in dietary quality may persist as the child develops [19,30,31]. One such study noted that parental stress was directly related to increased fast-food intake for children [19]. While we did not find a statistically significant difference in vegetable intake for children with parents who noted increased perceived stress, the magnitude and direction are similar to the associations noted in the previous study [19]. A recent study completed by Jansen et al. examined the relationship between parental stress regarding the COVID-19 pandemic and food practices in children [30]. This study noted that parents who reported higher parental stress had children who ate an increased amount of non-nutritive snacks, such as chips and ice cream [30]. In our multivariable analyses, we did not note an increase in added sugars or saturated fats with increased parental stress. However, we did find an increase in added sugars with HFI (while adjusting for parental stress). Unique to our study is the assessment of parental stress in the context of food insecurity.

We also showed that HFI was associated with lower total protein scores and parental stress with lower greens and beans intake. Other studies have supported the association of household food insecurity with decreased dietary variety [5,32]. Specifically, a study completed by Dave et al. noted that children who experienced greater food insecurity reported less fruit and vegetable intake [5]. Similarly, another study noted that children who had increased food security had a greater consumption of fruits and vegetables [32]. However, other additional research has noted no difference in dietary intake patterns for children aged 2–15 in food-secure or food-insecure households [33]. Inconsistencies in this literature may be attributed to differences in food insecurity categorization. Our study used a 2-item screen for measuring food insecurity, while others measured levels of food insecurity such as “marginal food security”, “low food security”, and “very low food security” [33]. This suggests there may be a difference in dietary quality based on the level of food insecurity. Future work should examine these associations in participants with multiple levels of household food insecurity.

Our study provides a unique contribution to the current body of literature as we identified how parental perceived stress and household food insecurity together contribute to the dietary variety of pre-school-aged children. Pediatric healthcare providers should be aware of these two factors, especially when they are seen together, and help address dietary quality in children. It is important to address dietary quality at a young age, as one study has shown that children of parents with increased stress, as measured by the PSS, had a steeper decline in their dietary quality over the course of one year [31]. This indicates that the effects of inadequate dietary variety can be long lasting and could ultimately negatively impact a child’s cognitive and physical development [34].

The limitations of this study should be noted. Most importantly, the small sample size may have prevented us from having adequate power to find statistically significant associations between variables. Future studies should examine these associations in a larger population of pre-school-aged children. The effect sizes noted in this study can be used for planning future larger-scale studies. This study included participants from four different pediatric clinics associated with one institution in North Carolina; the participants were also all English speaking. This could make the data less generalizable to other populations. Future research should be performed to include families from additional geographic locations to determine if our data are replicable in various geographic settings and with larger sample sizes. Additionally, the measures for parental stress and dietary recalls were based on parental self-report and thus could have some self-report bias. Parents may have misremembered their child’s dietary intake.

## 5. Conclusions

This sample of 24 racially and socioeconomically diverse parent–child dyads demonstrated that parental stress and HFI are associated with poorer dietary quality, especially with respect to decreased intake of greens and beans and increased intake of added sugars. To address this, pediatric offices should consider implementing regular screenings for parental stress and household food insecurity. In addition, providers should incorporate anticipatory guidance to improve dietary quality and refer patients to registered dietitians for counseling. Future studies should address whether interventions for food insecurity and parental stress improve children’s dietary quality.

## Figures and Tables

**Table 1:** Bivariate analyses of dietary quality components with household food insecurity.

Variable	Maximum Points	Total Sample Mean (SD)	Food Insecure Mean (SD)	Food Secure Mean (SD)	Effect Size	*p*-Value
HEI	100	55.2 (10.9)	51.0 (11.1)	55.8 (11.0)	0.44	0.5
Adequacy	60	32.2 (7.4)	28.6 (6.2)	32.8 (7.5)	0.56	0.4
Total vegetables	5	2.2 (1.5)	2.4 (1.0)	2.2 (1.5)	0.13	0.8
Greens and beans	5	1.2 (1.7)	1.3 (1.5)	1.2 (1.8)	0.06	0.9
Total fruit	5	3.4 (1.6)	2.9 (1.9)	3.4 (1.6)	0.31	0.6
Whole fruits	5	3.6 (1.5)	3.3 (2.8)	3.6 (1.3)	0.23	0.7
Whole grains	10	3.6 (3.5)	1.5 (1.3)	3.9 (2.3)	1.04	0.3
Total dairy	10	6.2 (2.9)	6.2 (2.5)	6.2 (2.9)	0.00	0.9
Total protein	5	4.4 (0.8)	4.2 (0.7)	4.5 (0.8)	0.38	0.6
Seafood and plant proteins	5	1.9 (2.0)	1.4 (1.6)	2.0 (2.1)	0.29	0.6
Fatty acids	10	5.7 (3.5)	5.4 (5.0)	5.7 (3.4)	0.09	0.9
Moderation	40	22.9 (4.6)	22.4 (5.1)	23.0 (4.6)	0.13	0.8
Sodium	10	4.5 (2.6)	3.7 (2.9)	4.7 (2.6)	0.38	0.6
Refined grains	10	5.5 (3.1)	4.4 (5.1)	5.7 (2.9)	0.45	0.5
Added sugars	10	6.7 (2.4)	9.2 (0.9)	6.3 (2.3)	1.26	0.04
Saturated fats	10	6.2 (2.7)	5.0 (2.4)	6.3 (2.7)	0.48	0.4

**Table 2:** Exploratory multivariate analyses examining the association of dietary quality components with household food insecurity and parental stress.

Separate Models with Dependent Variable of HEI Dietary Quality Component	Household Food Insecurity Beta (95%CI)	Parental Stress Beta (95%CI)	Income USD 20,000–USD 60,000 ^a^ Beta (95%CI)	Income USD 60,000 + ^a^ Beta (95%CI)	Child BMIz Beta (95%CI)	Male Sex Beta (95%CI)
HEI score (overall dietary quality)	−6.7	−3.9	−3.0	0.2	−0.5	0.6
Adequacy subscale	−5.7	−3.6	−1.1	−0.003	−0.6	1.0
Total vegetables	1.7	−1.2 +	2.3 *	1.0	0.2	−0.4
Greens and beans	0.4	−1.5 *	0.1	−0.2	0.2	−0.6
Total fruit	−1.0	1.1	−1.2	−0.8	0.3	0.6
Whole fruits	0.001	0.6	−0.2	0.6	0.1	0.1
Whole grains	−1.1	−1.0	0.6	4.0 +	−0.5	−1.1
Total dairy	0.4	1.3	−0.3	−0.2	0.3	−1.0
Total protein	−1.3 *	−0.4	−0.6	−0.7 +	−0.3 *	0.3
Seafood and plant proteins	−0.4	−1.3	1.0	−0.4	0.2	0.3
Fatty acids	−4.4 +	−1.3	−2.8	−3.2	−1.1 +	2.5 +
Moderation subscale	−1.0	−0.3	−1.9	0.02	0.1	−0.4
Sodium	1.9	1.2	1.1	3.5 *	0.8 +	0.8
Refined grains	−3.0	−0.4	−1.8	−1.7	−0.4	−0.8
Added sugars	4.1 *	−1.5	2.1	1.1	−0.05	−1.8 +
Saturated fats	−4.1 +	0.4	−3.3 +	−2.8 +	−0.2	1.4

^a^ Referent group for income categories was household income <USD 20,000; + denotes *p* < 0.1; * denotes *p* < 0.05.

## Data Availability

The data presented in this study are available on request from the corresponding author.
